# Transcriptomic Analyses of Pretreatment Tumor Biopsy Samples, Response to Neoadjuvant Chemoradiotherapy, and Survival in Patients With Advanced Rectal Cancer

**DOI:** 10.1001/jamanetworkopen.2022.52140

**Published:** 2023-01-20

**Authors:** Takashi Akiyoshi, Zhe Wang, Tomoko Kaneyasu, Osamu Gotoh, Norio Tanaka, Sayuri Amino, Noriko Yamamoto, Hiroshi Kawachi, Toshiki Mukai, Yukiharu Hiyoshi, Toshiya Nagasaki, Tomohiro Yamaguchi, Tsuyoshi Konishi, Yosuke Fukunaga, Tetsuo Noda, Seiichi Mori

**Affiliations:** 1 Department of Gastroenterological Surgery, Cancer Institute Hospital, Japanese Foundation for Cancer Research, Tokyo, Japan; 2 Project for Development of Innovative Research on Cancer Therapeutics, Cancer Precision Medicine Center, Japanese Foundation for Cancer Research, Tokyo, Japan; 3 Project for Development of Genomics-Based Cancer Medicine, Cancer Precision Medicine Center, Japanese Foundation for Cancer Research, Tokyo, Japan; 4 Division of Pathology, Cancer Institute, Japanese Foundation for Cancer Research, Tokyo, Japan; 5 Department of Colon and Rectal Surgery, The University of Texas MD Anderson Cancer Center, Houston; 6 Cancer Institute, Japanese Foundation for Cancer Research, Tokyo, Japan

## Abstract

**Importance:**

Neoadjuvant chemoradiotherapy (CRT) is the standard of care for advanced rectal cancer. Yet, estimating response to CRT remains an unmet clinical challenge.

**Objective:**

To investigate and better understand the transcriptomic factors associated with response to neoadjuvant CRT and survival in patients with advanced rectal cancer.

**Design, Setting, and Participants:**

A single-center, retrospective, case series was conducted at a comprehensive cancer center. Pretreatment biopsies from 298 patients with rectal cancer who were later treated with neoadjuvant CRT between April 1, 2004, and September 30, 2020, were analyzed by RNA sequencing. Data analysis was performed from July 1, 2021, to May 31, 2022.

**Exposures:**

Chemoradiotherapy followed by total mesorectal excision or watch-and-wait management.

**Main Outcomes and Measures:**

Transcriptional subtyping was performed by consensus molecular subtype (CMS) classification. Immune cell infiltration was assessed using microenvironment cell populations-counter (MCP-counter) scores and single-sample gene set enrichment analysis (ssGSEA). Patients with surgical specimens of tumor regression grade 3 to 4 or whose care was managed by the watch-and-wait approach for more than 3 years were defined as good responders.

**Results:**

Of the 298 patients in the study, 205 patients (68.8%) were men, and the median age was 61 (IQR, 52-67) years. Patients classified as CMS1 (6.4%) had a significantly higher rate of good response, albeit survival was comparable among the 4 subtypes. Good responders exhibited an enrichment in various immune-related pathways, as determined by ssGSEA. Microenvironment cell populations-counter scores for cytotoxic lymphocytes were significantly higher for good responders than nonresponders (median, 0.76 [IQR, 0.53-1.01] vs 0.58 [IQR, 0.43-0.83]; *P* < .001). Cytotoxic lymphocyte MCP-counter score was independently associated with response to CRT, as determined in the multivariable analysis (odds ratio, 3.81; 95% CI, 1.82-7.97; *P* < .001). Multivariable Cox proportional hazards regression analysis, including postoperative pathologic factors, revealed the cytotoxic lymphocyte MCP-counter score to be independently associated with recurrence-free survival (hazard ratio [HR], 0.38; 95% CI, 0.16-0.92; *P* = .03) and overall survival (HR, 0.16; 95% CI, 0.03-0.83; *P* = .03).

**Conclusions and Relevance:**

In this case series of patients with rectal cancer treated with neoadjuvant CRT, the cytotoxic lymphocyte score in pretreatment biopsy samples, as computed by RNA sequencing, was associated with response to CRT and survival. This finding suggests that the cytotoxic lymphocyte score might serve as a biomarker in personalized multimodal rectal cancer treatment.

## Key Points

**Question:**

What are the transcriptional profiles associated with response to neoadjuvant chemoradiotherapy (CRT) and survival in patients with advanced rectal cancer?

**Findings:**

In this case series of 298 patients with rectal cancer treated with neoadjuvant long-course CRT, RNA sequencing analysis of pretreatment biopsy samples showed that the cytotoxic lymphocyte score was independently associated with response to CRT. An association with both recurrence-free and overall survival was also noted.

**Meaning:**

The findings of this study suggest that the cytotoxic lymphocyte score computed from RNA sequencing in pretreatment tumor biopsy samples might serve as a biomarker in personalized multimodal rectal cancer treatment.

## Teaser

This case series examines transcriptomic factors associated with outcomes in patients with rectal cancer who received neoadjuvant chemoradiotherapy.

## Introduction

Neoadjuvant chemoradiotherapy (CRT) with total mesorectal excision is the standard of care for patients with locally advanced rectal cancer. Previous studies have noted that positive responses to CRT are associated with improved rates of long-term survival.^1,2^ However, CRT response varies from complete response to no tumor regression. Patients who show an excellent response to CRT may be candidates for the watch-and-wait approach, which avoids the definitive requirement for stoma and the resultant bowel or urogenital dysfunction associated with total mesorectal excision.^3,4,5,6^ However, more than 20% of patients treated in this way still experience local regrowth,^3^ presumably due to an inability to accurately diagnose pathologic response through current diagnostic methods.^3,7^ A better understanding of the molecular mechanisms underlying CRT response might improve the selection of candidate patients for organ-preserving treatment.

Various biomarkers are associated with response to CRT in rectal cancer, including the expression of p53,^8^ Ki67,^9^ thymidylate synthase,^10^ and CD133,^11^ as well as alterations in *KRAS*.^12^ Furthermore, several studies have reported gene expression signatures using microarray that predict responses to CRT in rectal cancer.^13,14^ Such biomarkers, however, have been neither consistently reproducible among studies nor validated for use in clinical practice.^15^

Previous work has highlighted the relevance of the immune tumor microenvironment for patient therapeutic responses and outcomes.^16^ In colon cancer, for example, immunoscores—derived from calculations of the densities of CD3^+^ and CD8^+^ T cells through immunohistochemistry—are highly reliable parameters for estimating recurrence.^17^ For patients with advanced rectal cancer, conventional immunohistochemical analyses have shown an association between higher density of tumor-infiltrating lymphocytes in pretreatment specimens and better response to CRT.^18,19,20^ Studies to date, however, have focused on a limited number of immune cell types and have failed to comprehensively investigate baseline proportions of immune cells and their relevance to CRT response and survival among patients with rectal cancer.^18,19,20^

Advances in next-generation sequencing technology and bioinformatics tools have resulted in quantitative transcriptomic data pertaining to various immune cell populations.^21,22^ Herein, we report an RNA sequencing-based transcriptomic analysis of pretreatment biopsy samples from 298 patients with advanced rectal cancer who were later treated with neoadjuvant CRT.

## Methods

### Patients and Tumor Samples

The current study was approved by the institutional review board of the Cancer Institute Hospital and was conducted in compliance with the Declaration of Helsinki^23^ and the reporting guideline for case series.^24^ Signed informed consent was obtained from all participants; no financial compensation was provided. We collected pretreatment, endoscopic, fresh-frozen biopsy tumor samples before patients commenced neoadjuvant CRT. Biopsies were stored at −150 °C. In our hospital, neoadjuvant CRT is indicated for clinical stage II/III low rectal cancer (ie, a tumor with the inferior border located below the peritoneal reflection). Pretreatment clinical stage was assessed using computed tomographic (CT) and pelvic magnetic resonance imaging. A flowchart for this study is shown in eFigure 1 in Supplement 1. From a total of 465 patients with rectal cancer without distant metastases who underwent conventional fluoropyrimidine-based long-course CRT without induction or consolidation chemotherapy between April 1, 2004, and September 30, 2020, a final cohort of 298 patients were selected. Samples were harvested prior to therapy and treated for RNA sequencing analysis.

### Tumor Regression Grade

Response to neoadjuvant CRT was retrospectively assessed histologically on formalin-fixed, surgically resected specimens according to the criteria of Dworak et al^25^ for tumor regression grade (TRG). The whole tumor area, including the regressed area, was sectioned at 4- to 5-mm intervals, and all cross-sections were evaluated, with the sample graded as a whole: TRG1, dominant tumor mass with obvious fibrosis; TRG2, dominantly fibrotic changes with few tumor cells; TRG3, very few (difficult to find microscopically) tumor cells in the fibrotic tissue; and TRG4, no viable tumor cells. Samples graded as TRG3 and TRG4 were grouped as good responders, whereas samples classified as TRG1 and TRG2 were grouped as nonresponders.

### RNA Preparation and Sequencing Analysis

Frozen biopsy tissues were cut into 10-μm-thick sections. Laser-capture microdissection with LMD7000 (Leica Microsystems) was used to enrich for cancer cells. Details on the RNA preparation and sequencing methods are available in the eMethods in Supplement 1.

### Bioinformatics Analyses for RNA-Sequencing Data

Significance analysis of microarrays (SAM) and receiver operating characteristic curve (ROC) analysis thresholds were used to identify genes that could distinguish between good responders and nonresponders, as per the following thresholds: SAM, *P* = .01; area under the curve, 0.60 or 0.40. For transcriptomic subtyping, consensus clustering was used to identify clusters corresponding to internal subgroups, using R with Bioconductor ConsensusClusterPlus.^26^ Based on variance in expression across samples, 3487 genes (top 10% variably expression selected by pvclust in R package) were selected and used for *K* means clustering with euclidean distance and a subsampling ratio of 0.8 for 1000 iterations. After correcting for batch effects with modified ComBat,^27^ CMS classification analysis was performed using a single-sample predictor in the R package CMSclassifier.^28^ To identify specific pathway enrichment associated with response to CRT in the transcriptome, single-sample gene set enrichment analysis (ssGSEA) was performed using R, version 4.1.0,^29^ with bioconductor gene set variation analysis and hallmark genes sets^30^ from the Molecular Signature DataBase, version 7.5.1. Enriched pathways were considered significant when SAM *q* values were less than 0.01. RNA-sequencing data were also analyzed using microenvironment cell populations-counter (MCP-counter), version 1.2.0, to infer which microenvironmental cell types infiltrated the tumor.^21^ We used ssGSEA to quantify the relative infiltration of 28 immune cell types.^22^ Cytolytic activity was estimated as the geometric mean of *GZMA* and *PRF1* in transcripts per kilobase million, with a 0.01 offset.^31^ Details on the gene expression database used in this study are available in the eMethods in Supplement 1.

### Statistical Analysis

Data analysis was performed from July 1, 2021, to May 31, 2022. Continuous variables were compared using the Mann-Whitney test, and categorical variables were analyzed with the Fisher exact test or the χ^2^ test. Overall survival (OS) was defined as the time from surgery to death from any cause, and recurrence-free survival (RFS) as the time from surgery to any recurrence. Among patients treated with the watch-and-wait approach, survival was calculated from the date of the decision to treat via the watch-and-wait approach. Survival curves were visualized using the Kaplan-Meier method with log-rank test. Univariable and multivariable logistic regression analyses were performed to evaluate the predictors of CRT response, and a Cox proportional hazards regression model was used to evaluate the predictors of OS and RFS. Variables with *P* values <.20 in the univariable analysis were examined by multivariable analysis. With 2-sided, unpaired testing, all *P* values <.05 were considered statistically significant. Analyses were performed using GraphPad Prism 7 software (GraphPad) or R software, version 4.1.0 (R Foundation for Statistical Computing).

## Results

### Transcriptional Subtyping, Response to CRT, and Survival

In the 298 patients included in the study, the median age was 61 years (IQR, 52-67 years); 205 patients (68.8%) were male, and 93 patients (31.2%) were female, with tumors at a median distance of 40 mm (IQR, 30-50 mm) from the anal verge. Patient clinicopathologic characteristics are summarized in the eTable in Supplement 1. Five patients were treated by the watch-and-wait approach, with no local regrowth or distant metastasis measured for more than 3 years as of April 13, 2022 (range, 3.0-4.9 years); these patients were included in the TRG4 group in subsequent analysis.

**Figure 1.**
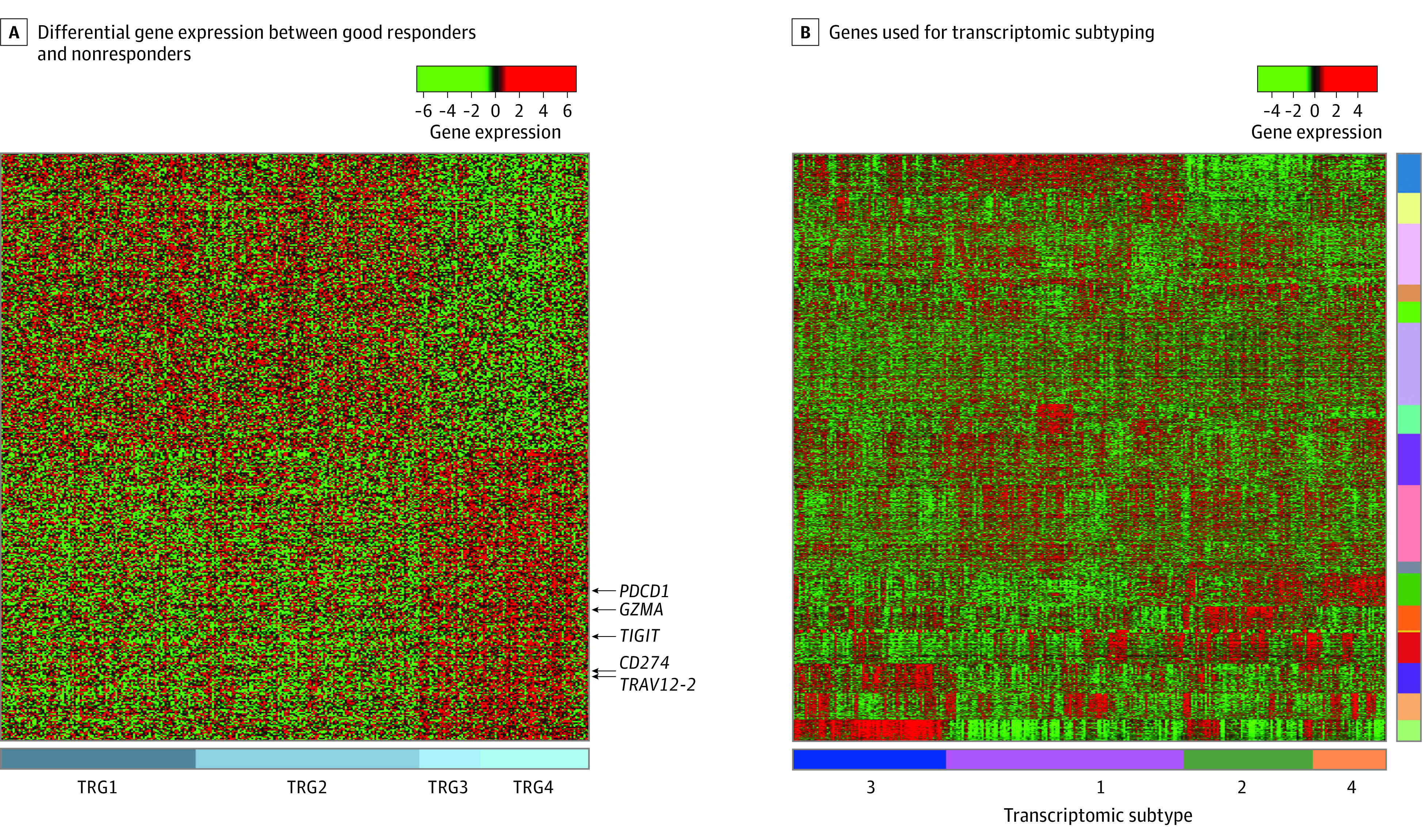
Differentially Expressed Genes Between Good Responders and Nonresponders and Gene Expression for Transcriptomic Subtypes A, Differential gene expression between good responders and nonresponders. The genes indicated are related to T cell effector function. B, Genes used for transcriptomic subtyping. Consensus clustering identified 4 transcriptional subgroups. Green indicates underexpression and red indicates overexpression of the genes. TRG indicates tumor regression grade. The labels for 17 gene clusters are shown on the right.

Binary comparisons of the gene expression values determined by SAM-ROC analyses revealed 216 differentially expressed genes for good responders and 218 for nonresponders (Figure 1A). Cytolytic effector *GZMA*; immune checkpoint molecules *PDCD1*, *TIGIT*, and *CD274*; and TCR α-chain *TRAV12-2* were highly expressed among the good responders. To examine whether the transcriptional subtype is associated with response to CRT and prognosis, *K* means consensus clustering was performed with 3487 genes; these genes were selected because of their highly variable expression across the cohort. Four transcriptomic subtypes were identified (Figure 1B), with the good responders distributed among the transcriptional subtypes (*P* = .03) (eFigure 2A in Supplement 1): the lowest proportion of good responders was found in subtype 1 (20.1%) and the highest in subtype 2 (40.0%). Recurrence-free survival was comparable among the 4 subtypes (eFigure 3A in Supplement 1).

Next, we performed CMS classification and found a significantly higher proportion of good responders in CMS1 (69.4%) compared with the other CMS subtypes (23.3%-37.5%) (eFigure 2B in Supplement 1). However, here again, RFS was comparable among the different CMS subtypes (eFigure 3B in Supplement 1). Together, these findings suggest that subtyping rectal cancer via consensus clustering or CMS criteria cannot be used to predict response to CRT or rates of survival.

**Figure 2.**
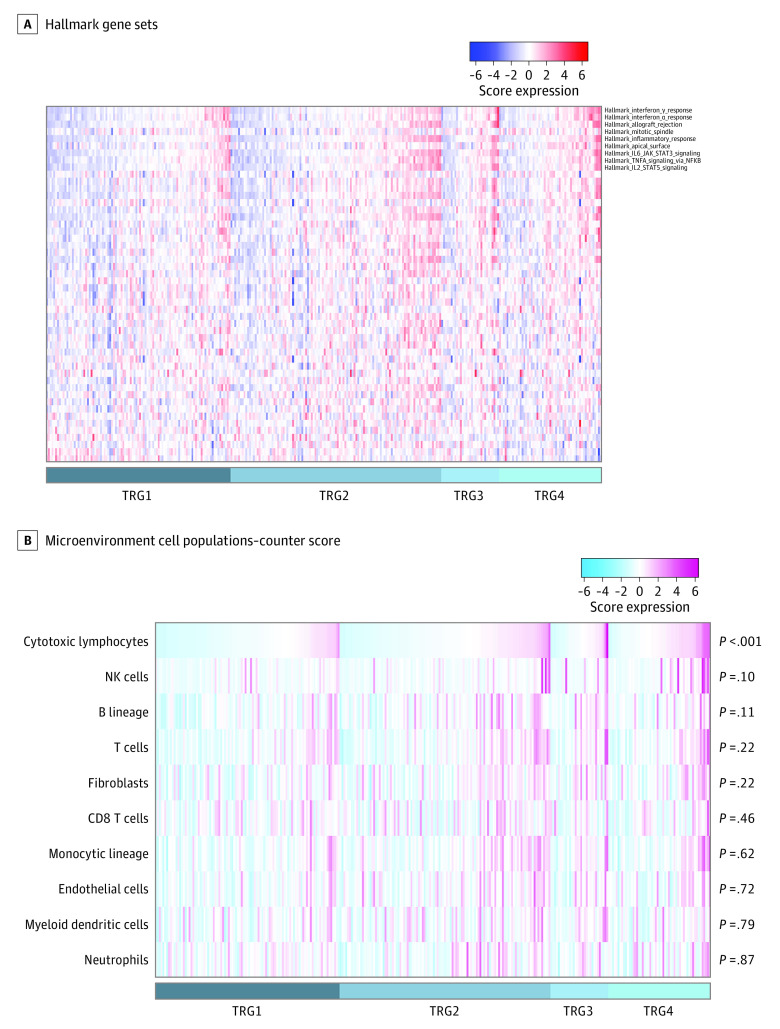
Hallmark Gene Sets and Microenvironment Cell Populations-Counter Score A, Fifty hallmark gene sets, distinctive between good responders and nonresponders, are ranked by their d-values by significance analysis of microarrays, with data shown as heatmaps. Blue indicates underexpression and red indicates overexpression of the gene sets. Gene sets of significance analysis of microarrays *q* value <.01 are indicated. B, Heatmap for microenvironment cell populations-counter scores. The *P* values were computed by Mann-Whitney tests and compare the scores between good responders and nonresponders. Cytotoxic lymphocytes showed the only significant difference (good responders > nonresponders). Blue indicates underexpression and violet-red indicates overexpression of the scores. NK indicates natural killer; TRG, tumor regression grade.

Finally, gene set analysis with ssGSEA using hallmark gene sets showed enrichment of gene sets associated with lymphocyte activation (hallmark_interferon_γ_response, hallmark_interferon_α_response, hallmark_inflammatory_response) (Figure 2A), suggesting the potential utility of immune cells in the tumor microenvironment in determining CRT response.

### Pretreatment Cytotoxic Lymphocyte Score, Response to CRT, and Survival

To further explore immune cells in the tumor microenvironment, we quantified 8 immune cell populations and 2 nonimmune stromal populations (endothelial cells and fibroblasts) by MCP-counter scoring. The MCP-counter scores for cytotoxic lymphocytes were significantly higher for good responders than nonresponders (median, 0.76 [IQR, 0.53-1.01] vs 0.58 [IQR, 0.43-0.83]; *P* < .001) (Figure 2B). Within each group, the MCP-counter scores for cytotoxic lymphocytes were significantly higher for TRG2 than TRG1 (median, 0.63 [IQR, 0.46-0.88] vs 0.53 [IQR, 0.37-0.73]; *P* = .03) and for TRG4 than TRG3 (median, 0.80 [IQR, 0.57-1.10] vs 0.62 [IQR, 0.39-0.86]; *P* = .03) (eFigure 4A in Supplement 1). The area under the curve of MCP-counter scores identifying good responders was 0.63. Cytolytic activity levels were also significantly higher for good responders than nonresponders (median, 1.83 [IQR, 0.72-2.94] vs 1.06 [IQR, 0.53-2.11]; *P* = .005), with levels higher for TRG2 than TRG1 (median, 1.28 [IQR, 0.68-2.60] vs 0.91 [IQR, 0.45-1.70]; *P* = .01); however, there was no significant difference between TRG4 and TRG3 (median, 2.01 [IQR, 0.75-3.63] vs 1.17 [IQR, 0.61-2.61]; *P* = .20) (eFigure 4B in Supplement 1). In the multivariable analysis using pretreatment clinicopathologic factors, clinical lymph node metastasis (odds ratio [OR], 0.53; 95% CI, 0.31-0.91; *P* = .02), radiation dose greater than or equal to 50 Gy (OR, 1.86; 95% CI, 1.03-3.36; *P* = .04), and MCP-counter scores for cytotoxic lymphocytes (OR, 3.81; 95% CI, 1.82-7.97; *P* < .001) were associated with response to CRT (Table 1). The MCP-counter scores for cytotoxic lymphocytes were also significantly higher for responders than nonresponders in GSE109057 (median, 3.60; IQR, 3.23-3.69 vs 3.28; IQR, 3.20-3.56; *P* = .01), GSE87211 (median, 6.22; IQR, 5.76-6.92 vs 5.93; IQR, 5.52-6.38; *P* = .009), and GSE45404 (median, 3.68; IQR, 3.40-3.76 vs 3.30; IQR, 3.16-3.58; *P* = .03) (eFigure 5 in Supplement 1) data sets.

**Table 1.** Univariable and Multivariable Analysis of Pretreatment Characteristics Associated With Tumor Regression Grade

Variable	Univariable analysis		Multivariable analysis	
	OR (95% CI)	*P* value	OR (95% CI)	*P* value
Age	1.02 (1.00-1.04)	.10	1.02 (1.00-1.05)	.07
Sex: male vs female	0.79 (0.46-1.34)	.38	NA	NA
Tumor distance from the anal verge: >40 vs ≤40 mm	0.99 (0.60-1.64)	.98	NA	NA
Time from CRT to surgery: >56 vs ≤56 d	1.70 (0.97-2.98)	.06	1.40 (0.77-2.58)	.27
Clinical T category: cT4 vs cT2/T3	0.71 (0.25-1.98)	.51	NA	NA
Clinical N category: positive vs negative	0.51 (0.31-0.85)	.009	0.53 (0.31-0.91)	.02
Pretreatment carcinoembryonic antigen: >5 vs ≤5 ng/mL	0.82 (0.48-1.40)	.46	NA	NA
Addition of oxaliplatin to CRT: yes vs no	1.71 (0.67-4.34)	.26	NA	NA
Radiation dose: ≥50 vs 45 Gy	1.94 (1.12-3.37)	.02	1.86 (1.03-3.36)	.04
Histologic characteristic: well/moderate vs others	0.39 (0.12-1.24)	.11	0.61 (0.16-2.39)	.48
Cytotoxic lymphocyte score	3.92 (1.95-7.87)	<.001	3.81 (1.82-7.97)	<.001

Abbreviations: CRT, chemoradiotherapy; NA, not applicable; OR, odds ratio.

**Figure 3.**
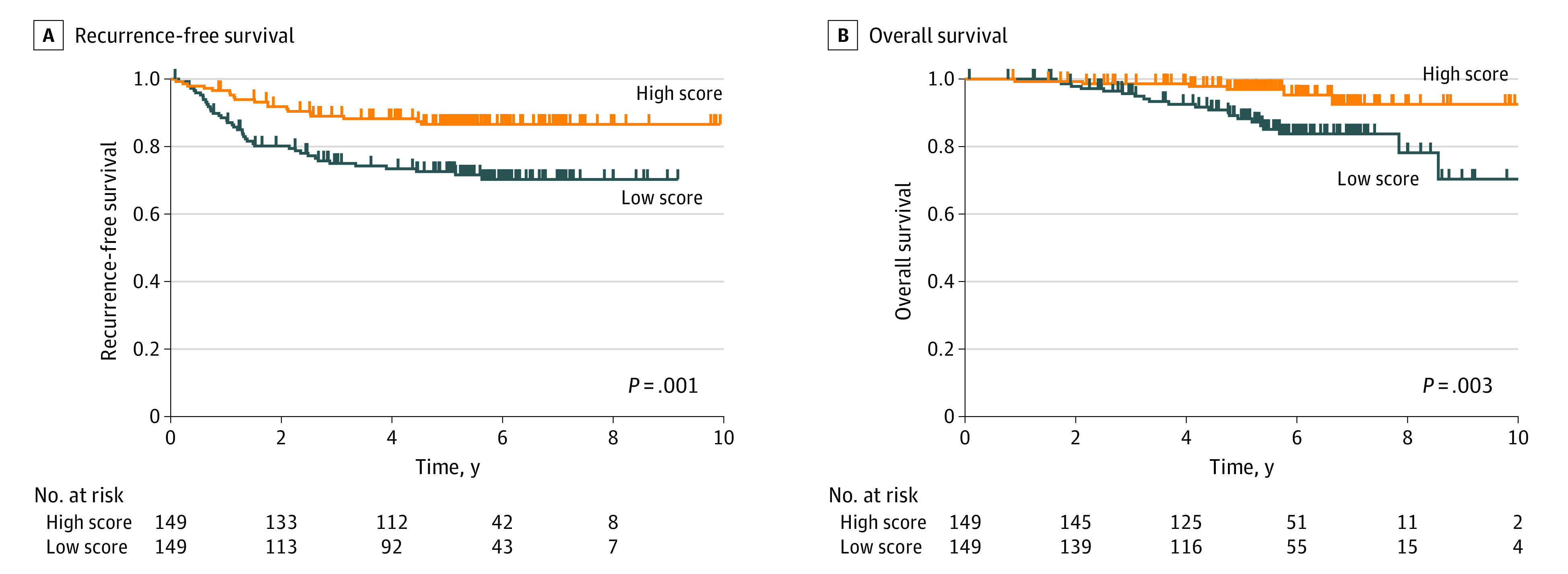
Microenvironment Cell Populations-Counter Scores of Cytotoxic Lymphocytes Recurrence-free survival (A) and overall survival (B) by high or low microenvironment cell populations-counter scores of cytotoxic lymphocytes. The *P* values were computed by log-rank test.

The median follow-up period for survivors who underwent surgery was 5.6 years (IQR, 4.9-6.7 years) as of April 13, 2022. When patients were divided into 2 groups based on the median value (high vs low MCP-counter scores for cytotoxic lymphocytes), RFS (5-year RFS of 86.6% vs 72.6%; *P* = .001) (Figure 3A) and OS (5-year OS of 97.0% vs 88.3%; *P* = .003) (Figure 3B) were significantly better for the group with the higher scores. To examine whether MCP-counter scores for cytotoxic lymphocytes would be associated with RFS and OS independent of pathologic stage, we performed a multivariable analysis using pretreatment and postoperative clinicopathologic factors in 293 patients who underwent surgery. We found that time from CRT to surgery longer than 56 days (hazard ratio [HR], 2.30; 95% CI, 1.34-3.95; *P* = .003), MCP-counter scores for cytotoxic lymphocytes (HR, 0.38; 95% CI, 0.16-0.92; *P* = .03), ypT3/4 (HR, 2.75; 95% CI, 1.42-5.34; *P* = .003), ypN-positive samples (HR, 3.71; 95% CI, 1.96-7.02; *P* < .001), and no adjuvant chemotherapy (HR, 2.01; 95% CI, 1.08-3.74; *P* = .03) were associated with RFS (Table 2). In addition, MCP-counter scores for cytotoxic lymphocytes (HR, 0.16; 95% CI, 0.03-0.83; *P* = .03) and ypN-positive samples (HR, 2.86; 95% CI, 1.22-6.71; *P* = .02) were associated with OS (Table 2). We also found higher MCP-counter scores for cytotoxic lymphocytes to be associated with higher levels of common immune checkpoint genes (*TIGIT*, *PDCD1*, *LAG3*, *CTLA4*, and *CD274*) (eFigure 6 in Supplement 1).

**Table 2.** Univariable and Multivariable Analysis of Clinicopathologic Factors Associated With Recurrence-Free Survival and Overall Survival

Variable	Recurrence-free survival				Overall survival			
	Univariable analysis		Multivariable analysis		Univariable analysis		Multivariable analysis	
	HR (95% CI)	*P* value	HR (95% CI)	*P* value	HR (95% CI)	*P* value	HR (95% CI)	*P* value
Age	1.00 (0.97-1.02)	.71	NA	NA	1.03 (0.99-1.07)	.13	1.01 (0.97-1.06)	.50
Sex: male vs female	1.19 (0.68-2.09)	.54	NA	NA	1.32 (0.56-3.12)	.53	NA	NA
Tumor distance from the anal verge: >40 vs ≤40 mm	0.72 (0.43-1.21)	.21	NA	NA	1.25 (0.59-2.67)	.56	NA	NA
Time from CRT to surgery: >56 vs ≤56 d	2.42 (1.43-4.09)	.001	2.30 (1.34-3.95)	.003	1.28 (0.51-3.18)	.60	NA	NA
Clinical T category: cT4 vs cT2/T3	1.75 (0.79-3.84)	.17	1.19 (0.53-2.68)	.67	3.00 (1.13-7.96)	.03	2.44 (0.89-6.72)	.08
Clinical N category: positive vs negative	1.16 (0.70-1.94)	.57	NA	NA	1.52 (0.68-3.39)	.30	NA	NA
Pretreatment carcinoembryonic antigen: >5 vs ≤5 ng/mL	1.31 (0.78-2.20)	.30	NA	NA	2.11 (0.99-4.48)	.05	1.48 (0.67-3.29)	.33
Addition of oxaliplatin to CRT: yes vs no	0.67 (0.21-2.13)	.49	NA	NA	0.00 (0.00-infinity)	>.99	NA	NA
Radiation dose: ≥50 vs 45 Gy	1.24 (0.73-2.11)	.42	NA	NA	0.68 (0.32-1.47)	.33	NA	NA
Histologic characteristic: well/moderate vs others	0.55 (0.20-1.52)	.25	NA	NA	1.49 (0.20-11.25)	.70	NA	NA
Cytotoxic lymphocyte score	0.28 (0.11-0.70)	.007	0.38 (0.16-0.92)	.03	0.11 (0.02-0.58)	.009	0.16 (0.03-0.83)	.03
ypT category: ypT3/4 vs ypT1/2	4.79 (2.67-8.59)	<.001	2.75 (1.42-5.34)	.003	2.70 (1.21-6.01)	.02	1.20 (0.45-3.25)	.72
ypN category: positive vs negative	4.03 (2.41-6.71)	<.001	3.71 (1.96-7.02)	<.001	3.56 (1.65-7.72)	<.001	2.86 (1.22-6.71)	.02
Tumor regression grade: 3/4 vs 1/2	0.22 (0.09-0.54)	.001	0.47 (0.17-1.28)	.14	0.35 (0.11-1.16)	.08	0.64 (0.15-2.61)	.53
Adjuvant chemotherapy: no vs yes	0.64 (0.38-1.06)	.08	2.01 (1.08-3.74)	.03	0.76 (0.36-1.63)	.48	NA	NA

Abbreviations: CRT, chemoradiotherapy; HR, hazard ratio; NA, not applicable.

Finally, we used ssGSEA to estimate associations among 28 subpopulations of immune cells and CRT response. The enrichment scores for 7 subpopulations were significantly higher among good responders than nonresponders (SAM *q* < 0.05) (eFigure 7 and eFigure 8 in Supplement 1). These 7 subpopulations were 4 subpopulations of cytotoxic cells (effector memory CD8^+^ T cells, natural killer [NK] T cells, NK cells, and activated CD8^+^ T cells) and 3 subpopulations of CD4^+^ T cells (activated CD4^+^ T cells, type 2 T helper cells, and type 1 T helper cells).

## Discussion

To our knowledge, this is the largest study of RNA sequencing analysis using pretreatment biopsy samples from patients with advanced rectal cancer. We found preexisting cytotoxic lymphocytes to be associated with response to CRT and survival. The same association was found in other publicly available data sets using microarray, despite the use of a different microarray platform and different set of criteria for responders vs nonresponders among the data sets.

Some studies have used microarray technology to report the differential expression of genes between responders and nonresponders as a predictive biomarker of CRT response in rectal cancer^32^; however, none of these previous gene signatures has been reproducible.^33,34^ The present study identified 4 transcriptomic subtypes based on highly variable genes identified through consensus clustering. Good responders distributed differently among the transcriptional subtypes, but these subtypes were not associated with survival, suggesting that transcriptional subtyping does not allow for a prediction of response to CRT or survival in rectal cancer. Notably, CMS1, characterized by hypermutated, microsatellite unstable, and strong immune activation,^28^ was associated with good responders. Consistent with our findings, previous studies have reported that microsatellite unstable rectal cancer is associated with an increased rate of pathologic complete response.^18,35,36^ However, CMS1 is rarely observed in rectal cancer (3% in the original study^28^ and 6% in the present study), and there were no significant differences in response to CRT or survival among CMS2 to 4 or unclassified subtypes. As such, subtyping of rectal cancer by CMS criteria is not useful for the estimation of response to CRT or survival. Our findings agree with those of Nicolas et al,^37^ who noted that CMS classification in 212 patients with rectal cancer treated with CRT was not associated with survival.

In our study, good responders exhibited enrichment in various immune-related pathways (interferon [IFN]-γ and IFN-α responses, allograft rejection, interleukin-6 signal transducer and activator of transcription 3 signaling during the acute phase response, and inflammation). Interferon-γ is primarily produced by tumor-infiltrating CD8^+^ T cells and NK cells in colorectal cancer,^38^ and IFN-α boosts the function of CD8^+^ T cells and NK cells by increasing the expression levels of granzyme B and perforin.^39^ Similarly, we found significantly higher proportions of cytotoxic lymphocytes from MCP-counter analyses^21^ and measurements of cytolytic activity based on the transcript levels of *GZMA* and *PRF1*^31^ among good responders compared with nonresponders. Cytotoxic lymphocytes from MCP-counter analyses were also significantly different between the groups according to the extent of tumor regression, suggesting that cytotoxic lymphocyte MCP-counter score is an accurate predictive biomarker of the response to CRT in patients with rectal cancer. Concordant results were observed with ssGSEA analyses, which revealed that cytotoxic cells (effector memory CD8^+^ T cells, NK T cells, NK cells, and activated CD8^+^ T cells) and some subpopulations of CD4^+^ T cells were significantly higher among good responders than nonresponders; these CD4+ T cells play important roles in maintaining cytotoxic response.^40^ We have previously reported pretreatment tumor-infiltrating CD8^+^ cell density—not stromal cell density—to be correlated with high sensitivity to CRT through immunohistochemical analyses.^18^ However, it is not possible to obtain a comprehensive description of immune cells or an evaluation of immune cell functional state using immunohistochemistry. Ruffell and colleagues^41^ reported minimal granzyme B staining in treatment-naive breast cancer, even in areas with high numbers of CD8^+^ T cells, suggesting that most CD8^+^ T cells might not be in an activated state. In contrast, our findings provide evidence that functionally active cytotoxic lymphocytes at baseline are associated with high sensitivity to CRT and better survival in patients with rectal cancer.

We further noted an association between high expression of cytotoxic lymphocytes and immunosuppressive factors, such as *PDCD1* and *CD274*. These findings are consistent with a previous report by Rooney et al,^31^ which showed a positive correlation between cytolytic activity and the expression of immune-checkpoint genes in various types of cancer. These results suggest that the higher cytotoxic lymphocyte activity is accompanied by counter-regulatory activities that might limit the immune response. However, others have reported that high programmed cell death-1 expression within tumor-infiltrating lymphocytes is correlated with improved survival in colorectal cancer.^42,43^ As such, a high expression of immune-checkpoint genes at baseline might reflect an elevated effector function and might indicate high sensitivity to CRT. There has been an increasing number of clinical trials in recent years testing new combinations of radiation and immune-checkpoint inhibitors in rectal cancer.^44,45^ Indeed, in patients with rectal cancer, high programmed cell death-1 expression in CD8^+^ T cells is significantly correlated with high sensitivity to CRT with consolidation nivolumab.^44^ Likewise, among patients with non–small cell lung cancer, high programmed cell death-1 expression in CD8^+^ T cells is associated with tumor recognition and patient responses to immune checkpoint inhibitors.^46^ As such, high proportions of cytotoxic lymphocytes might thus serve as a predictive marker for CRT efficacy when delivered with immune checkpoint inhibitors.

### Strengths and Limitations

The strengths of this study are the long-term follow-up time for assessing treatment response followed with standard long-course neoadjuvant CRT, as well as the large case series. To our knowledge, this is the largest study to date using RNA sequencing in rectal cancer.

The study has limitations. First, the design was retrospective and its setting was a single cancer center in Japan. Second, few patients were treated by the watch-and-wait approach, patients treated with total neoadjuvant therapy were not included, and clinical responses after CRT were not analyzed. The internal and external validation study using the case series of patients treated with total neoadjuvant therapy or the watch-and-wait approach would be necessary. Third, the performance of cytotoxic lymphocyte scores identifying good responders was not enough, with the area under the curve of 0.63. Considering the other score (eg, hallmark_mitotic_spindle score by ssGSEA analysis) might improve the performance identifying good responders and needs to be further investigated.

## Conclusions

In this case series study of patients with rectal cancer treated with neoadjuvant long-course CRT, RNA sequencing of pretreatment biopsy samples highlighted the relevance of cytotoxic lymphocytes at baseline in response to CRT and survival. This finding suggests that analysis of cytotoxic lymphocytes might serve as a new marker for patients with rectal cancer.

## Notes

Supplement 1. **eTable.** Clinicopathological Characteristics **eFigure 1.** Flow Chart **eFigure 2.** Proportions of Good Responders and Nonresponders for Transcriptomic or Consensus Molecular Subtypes **eFigure 3.** Kaplan-Meier Recurrence-Free Survival Curve by Transcriptomic or Consensus Molecular Subtypes **eFigure 4.** Scatter Plots of Microenvironment Cell Populations-Counter (MCP-Counter) Scores of Cytotoxic Lymphocytes and Cytolytic Activity by Tumor Regression Grade **eFigure 5.** Scatter Plots of Microenvironment Cell Populations-Counter (MCP-Counter) Scores of Cytotoxic Lymphocytes in External Datasets **eFigure 6.** Scatter Plots of Transcript Level of Immune Checkpoint Genes by High or Low Microenvironment Cell Populations-Counter (MCP-Counter) Scores of Cytotoxic Lymphocytes **eFigure 7.** Heatmap for Single-Sample Gene Set Enrichment Analysis (ssGSEA) Scores of 28 Immune Subpopulations **eFigure 8.** Scatter Plots of Single-Sample Gene Set Enrichment Analysis (ssGSEA) Scores for the 7 Immune Subpopulations Showing a Significant Difference Between Good Responders and Nonresponders **eMethods.** Detailed Methods
eReferences

Supplement 2.
Data Sharing Statement
