# Supplementary material for: Transcriptomic Analyses of Pretreatment Tumor Biopsy Samples, Response to Neoadjuvant Chemoradiotherapy, and Survival in Patients With Advanced Rectal Cancer
Source: JAMA Netw Open. 2023 Jan 20;6(1):e2252140. doi: 10.1001/jamanetworkopen.2022.52140 (PMC9860531; doi:10.1001/jamanetworkopen.2022.52140)
Supplement: Supplement 1. — eTable. Clinicopathological Characteristics eFigure 1. Flow Chart eFigure 2. Proportions of Good Responders and Nonresponders for Transcriptomic or Consensus Molecular Subtypes eFigure 3. Kaplan-Meier Recurrence-Free Survival Curve by Transcriptomic or Consensus Molecular Subtypes eFigure 4. Scatter Plots of Microenvironment Cell Populations-Counter (MCP-Counter) Scores of Cytotoxic Lymphocytes and Cytolytic Activity by Tumor Regression Grade eFigure 5. Scatter Plots of Microenvironment Cell Populations-Counter (MCP-Counter) Scores of Cytotoxic Lymphocytes in External Datasets eFigure 6. Scatter Plots of Transcript Level of Immune Checkpoint Genes by High or Low Microenvironment Cell Populations-Counter (MCP-Counter) Scores of Cytotoxic Lymphocytes eFigure 7. Heatmap for Single-Sample Gene Set Enrichment Analysis (ssGSEA) Scores of 28 Immune Subpopulations eFigure 8. Scatter Plots of Single-Sample Gene Set Enrichment Analysis (ssGSEA) Scores for the 7 Immune Subpopulations Showing a Significant Difference Between Good Responders and Nonresponders eMethods. Detailed Methods eReferences [file jamanetwopen-e2252140-s001.pdf]

Supplementary Online Content

Akiyoshi T, Wang Z, Kaneyasu T, et al. Transcriptomic analyses of pretreatment tumor biopsy samples, response to neoadjuvant chemoradiotherapy, and survival in patients with advanced rectal cancer. *JAMA Netw Open*. 2023;6(1):e2252140. doi:10.1001/jamanetworkopen.2022.52140

eTable. Clinicopathological Characteristics

eFigure 1. Flow Chart

eFigure 2. Proportions of Good Responders and Nonresponders for Transcriptomic or Consensus Molecular Subtypes

eFigure 3. Kaplan-Meier Recurrence-Free Survival Curve by Transcriptomic or Consensus Molecular Subtypes

eFigure 4. Scatter Plots of Microenvironment Cell Populations-Counter (MCP-Counter) Scores of Cytotoxic Lymphocytes and Cytolytic Activity by Tumor Regression Grade

eFigure 5. Scatter Plots of Microenvironment Cell Populations-Counter (MCP-Counter) Scores of Cytotoxic Lymphocytes in External Datasets

eFigure 6. Scatter Plots of Transcript Level of Immune Checkpoint Genes by High or Low Microenvironment Cell Populations-Counter (MCP-Counter) Scores of Cytotoxic Lymphocytes

eFigure 7. Heatmap for Single-Sample Gene Set Enrichment Analysis (ssGSEA) Scores of 28 Immune Subpopulations

eFigure 8. Scatter Plots of Single-Sample Gene Set Enrichment Analysis (ssGSEA) Scores for the 7 Immune Subpopulations Showing a Significant Difference Between Good Responders and Nonresponders

eMethods. Detailed Methods

eReferences

This supplementary material has been provided by the authors to give readers additional information about their work.

© 2023 Akiyoshi T et al. *JAMA Network Open.*

eTable. Clinicopathological characteristics

Characteristics	No. of patients[1]
Sex	
Male	205 (68.8%)
Female	93 (31.2%)
Age (years)	61 (52–67)
Tumor distance from the anal verge (mm)	40 (30–50)
Clinical T category	
T2	3 (1.0%)
T3	273 (91.6%)
T4	22 (7.4%)
Clinical N category	
N0	138 (46.3%)
N+	160 (53.7%)
Histological type	
Well/moderate	286 (96.0%)
Others	12 (4.0%)
Pre-treatment CEA (ng/mL)	
≤5	195 (65.4%)
>5	103 (34.6%)
CRT regimen	
Fluoropyrimidine-based	278 (93.3%)
Addition of oxaliplatin	20 (6.7%)
Radiation dose (Gy)	
45	111 (37.2%)
50-50.4	187 (62.8%)
Interval from the completion of CRT to surgery or decision of WW (days)	49 (44–56)
Operative procedure	
Sphincter preserving	216 (72.5%)
Sphincter non-preserving	77 (25.8%)
WW	5 (1.7%)
ypT category	
ypT0	50 (17.1%)
ypTis	4 (1.4%)

© 2023 Akiyoshi T et al. *JAMA Network Open.*

ypT1	19 (6.5%)
ypT2	92 (31.4%)
ypT3	122 (41.6%)
ypT4	6 (2.0%)
ypN category	
ypN0	212 (72.4%)
ypN+	81 (27.6%)
R status	
R0	289 (98.6%)
R1	4 (1.4%)
Tumor regression grade (TRG)	
TRG1	99 (33.8%)
TRG2	113 (38.6%)
TRG3	31 (10.6%)
TRG4	50 (17.1%)
Adjuvant chemotherapy	116 (40.0%)

[1]Data are number (%) or median (IQR).

Abbreviations: CEA, carcinoembryonic antigen; CRT, chemoradiotherapy; WW, watch and wait.

© 2023 Akiyoshi T et al. *JAMA Network Open.*

eFigure 1. Flow chart

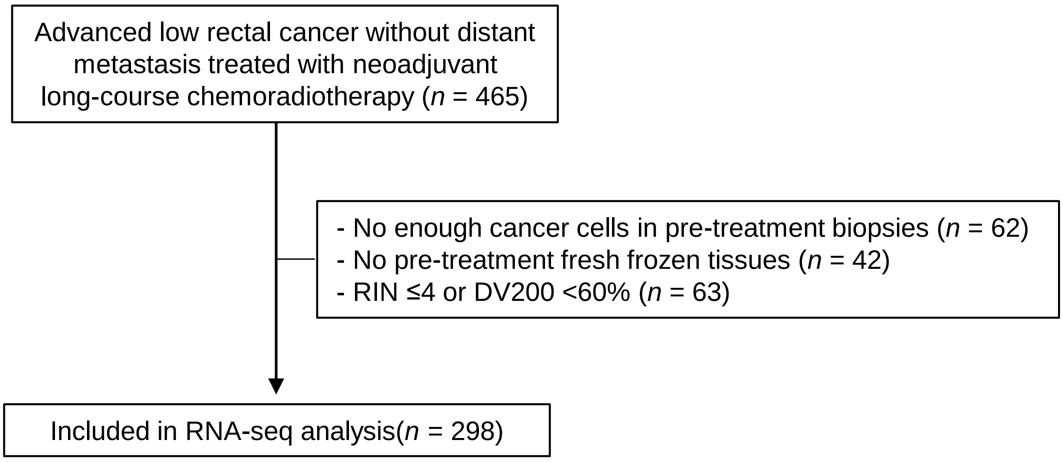

Of the 465 patients treated with neoadjuvant long-course chemoradiotherapy recruited, 298 patients were included in the RNA sequencing. RIN, RNA integrity number.

© 2023 Akiyoshi T et al. *JAMA Network Open.*

eFigure 2. Proportions of good responders and nonresponders for transcriptomic or consensus molecular subtypes.

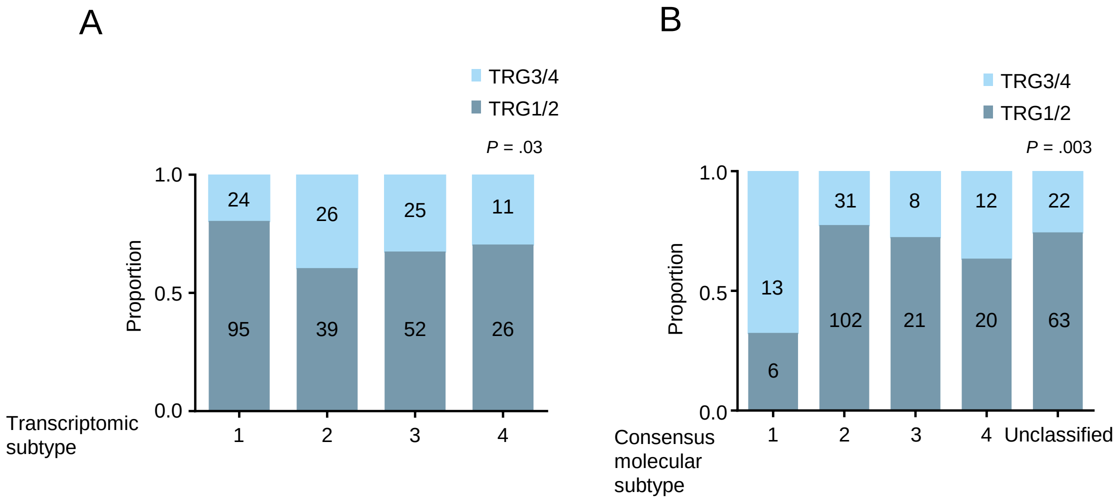

(A) Proportions of good responders and non-responders for transcriptomic subtypes. Sample number is also shown on the stacked bar plots. The *P*-value was computed by χ^2 test. (B) Proportions of good responders and non-responders for consensus molecular subtypes. Sample number is also shown on the stacked bar plots. The *P*-value was computed by χ^2 test.

© 2023 Akiyoshi T et al. *JAMA Network Open.*

eFigure 3. Kaplan–Meier recurrence-free survival curve by transcriptomic or consensus molecular subtypes.

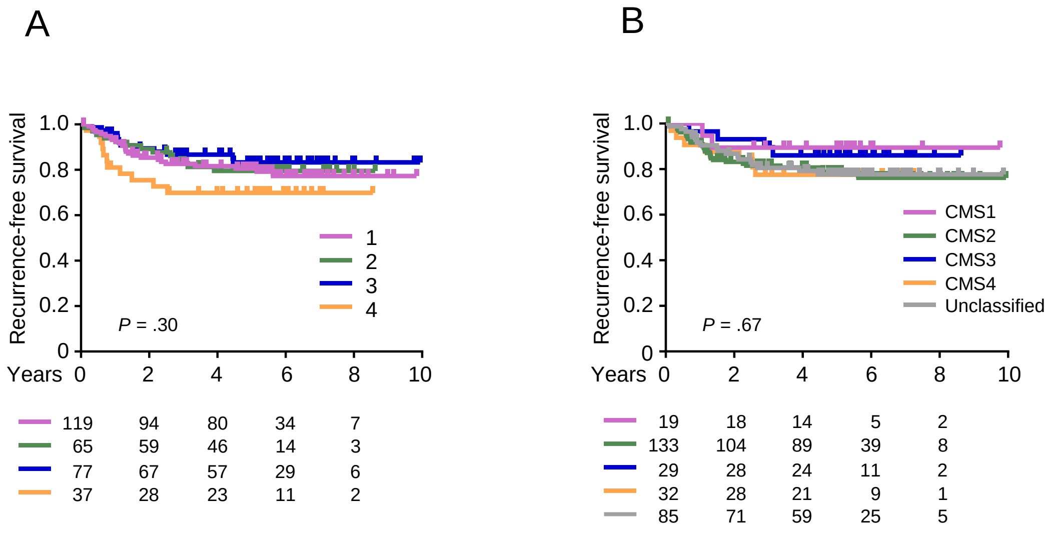

(A) Kaplan–Meier recurrence-free survival curve by transcriptomic subtypes. The *P*-value was computed by log-rank test. (B) Kaplan–Meier recurrence-free survival curve by consensus molecular subtypes. The *P*-value was computed by log-rank test.

© 2023 Akiyoshi T et al. *JAMA Network Open.*

eFigure 4. Scatter plots of Microenvironment Cell Populations-counter (MCP-counter) scores of cytotoxic lymphocytes and cytolytic activity by tumor regression grade.

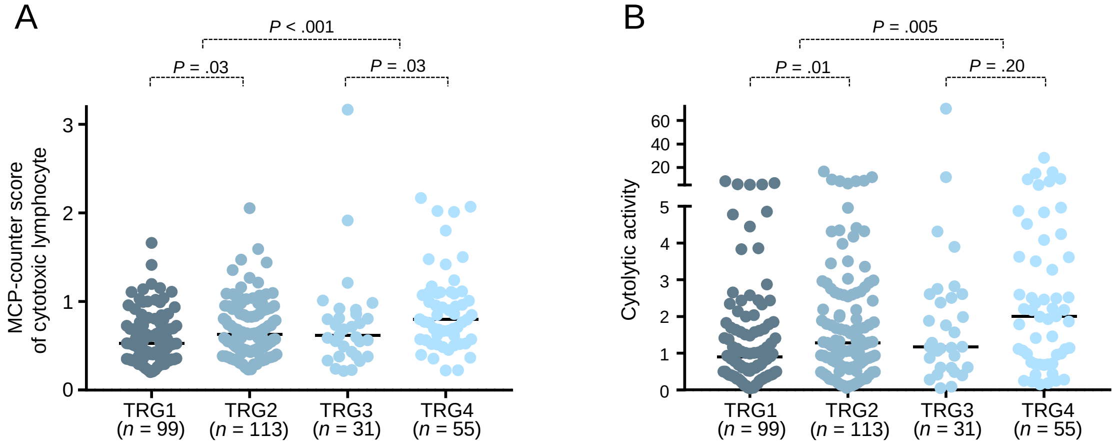

(A) Scatter plots of MCP-counter scores of cytotoxic lymphocytes by tumor regression grade (TRG). The median value is represented by a horizontal line. The *P*-values were computed by Mann–Whitney U-tests. (B) Scatter plots of cytolytic activity by TRG. The median value is represented by a horizontal line. The *P*-values were computed by Mann–Whitney U-tests.

© 2023 Akiyoshi T et al. *JAMA Network Open.*

eFigure 5. Scatter plots of Microenvironment Cell Populations-counter (MCP-counter) scores of cytotoxic lymphocytes in external datasets.

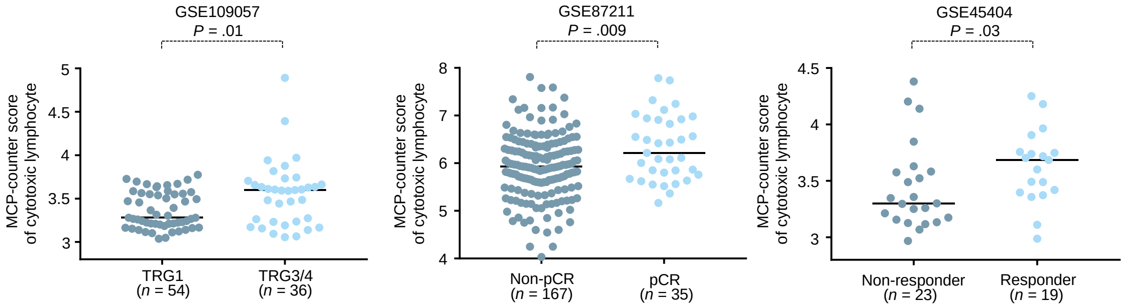

Scatter plots of MCP-counter scores for cytotoxic lymphocytes in GSE109057 (left panel), GSE87211 (middle panel), and GSE45404 (right panel) as a function of response to CRT. The median value is represented by a horizontal line. The *P*-values were computed by Mann–Whitney U-tests.

© 2023 Akiyoshi T et al. *JAMA Network Open.*

eFigure 6. Scatter plots of transcript level of immune checkpoint genes by high or low Microenvironment Cell Populations-counter (MCP-counter) scores of cytotoxic lymphocytes.

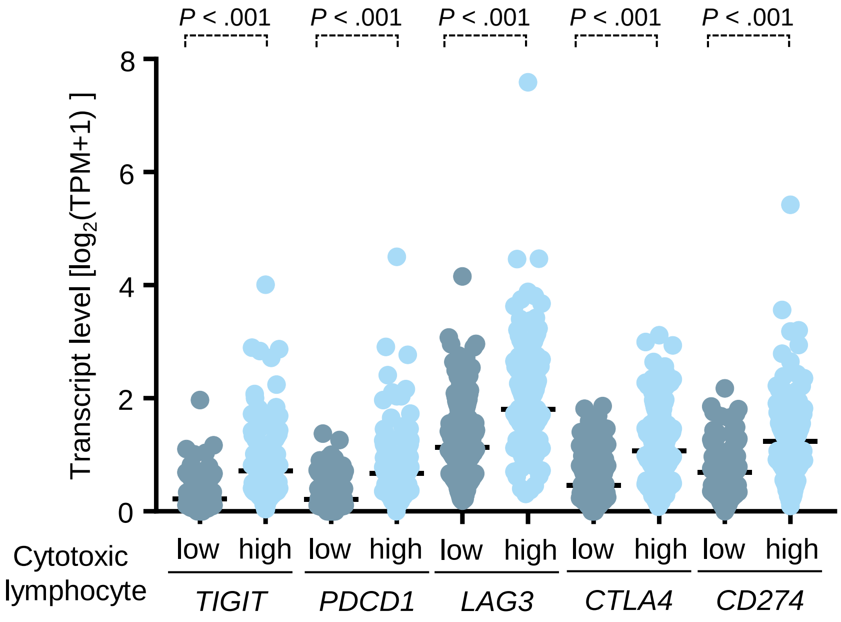

Scatter plots of transcript level of immune checkpoint genes (*TIGIT*, *PDCD1*, *LAG3*, *CTLA4*, and *CD274*) by high or low MCP-counter scores of cytotoxic lymphocytes. The *P*-values were computed by Mann–Whitney U-tests.

© 2023 Akiyoshi T et al. *JAMA Network Open.*

eFigure 7. Heatmap for single-sample gene set enrichment analysis (ssGSEA) scores of 28 immune subpopulations.

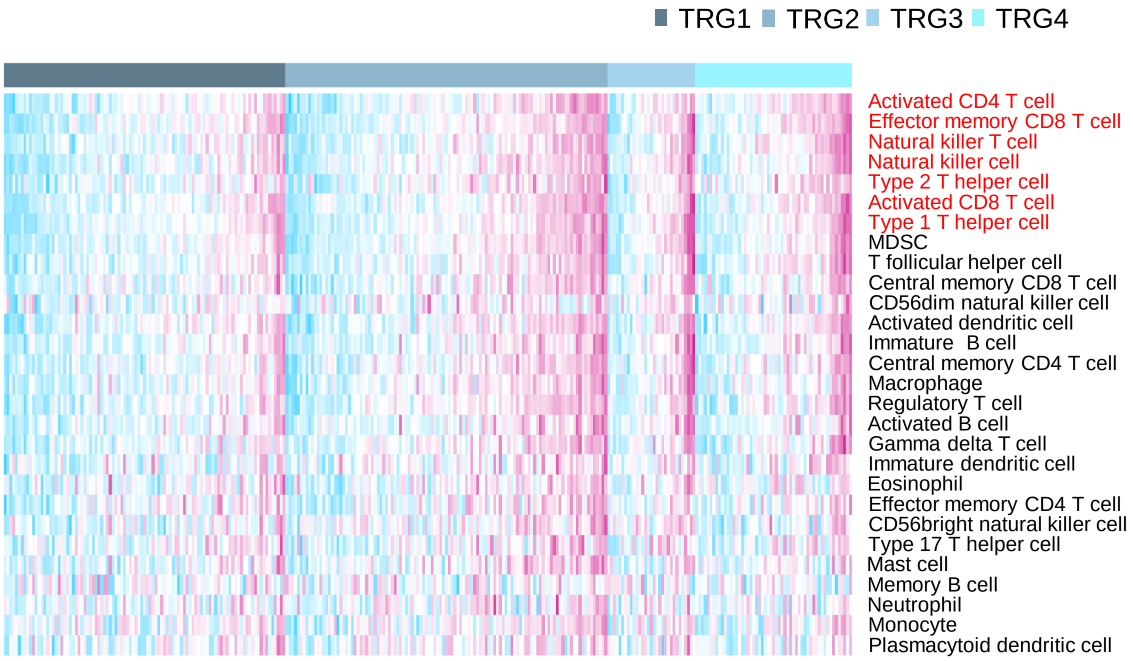

Heatmap for ssGSEA scores of 28 immune subpopulations ranked by their d-values, as determined using SAM. Immune subpopulations showing a significant difference (SAM q-value < .05) between good responders and non-responders are indicated in red (good responders > non-responders). Sky blue and violet-red colors indicate under- and over-expression of the scores, respectively.

© 2023 Akiyoshi T et al. *JAMA Network Open.*

eFigure 8. Scatter plots of single-sample gene set enrichment analysis (ssGSEA) scores for the 7 immune subpopulations showing a significant difference between good responders and nonresponders.

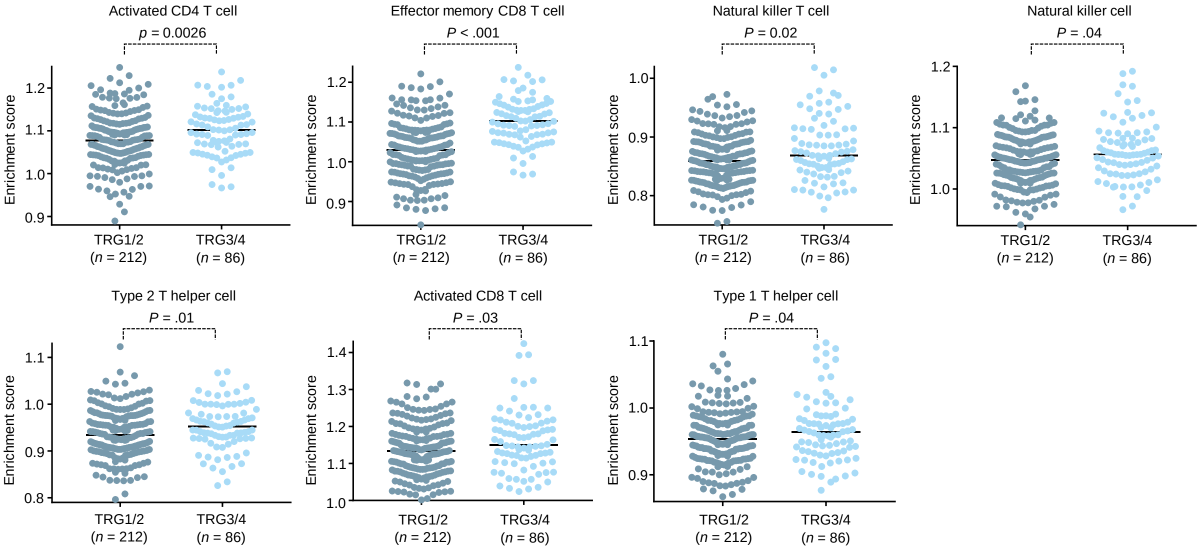

The *P*-values were computed by Mann–Whitney U-tests.

© 2023 Akiyoshi T et al. *JAMA Network Open.*

eMethods. Detailed Methods

RNA preparation and sequencing analysis

Frozen biopsy tissues were cut into 10-μm-thick sections. Laser-capture microdissection with LMD7000 (Leica Microsystems, Bensheim, Germany) was used to enrich for cancer cells. Total RNA from fresh-frozen tumor samples was extracted using the AllPrep DNA/RNA Mini Kit (Qiagen) according to the manufacturer's instructions. RNA quality and quantity were checked with the NanoDrop 2000 (ThermoFisher) and Agilent 2100 Bioanalyzer (Agilent). RNA samples that passed the criteria for RNA purity (RNA integrity number [>4] and DV200 [≥60]) were further processed for RNA sequencing. mRNA libraries were constructed using the TruSeq RNA Access Library Prep Kit (Illumina), with the starting amount of RNA determined as per the manufacturer's instructions. Libraries were sequenced as 101+8+8+101 bp with a dual-indexed run on an Illumina HiSeq2500. RNA reads were aligned to the GRCh37 reference genome. Gene expression values were computed as Transcripts Per Kilobase Million (TPMs) by RSEM-1.3.0,[1] mapped by bowtie2-2.2.3, and annotated by ENSEMBL release 75. To avoid an infinite value, TPMs were transformed by log2 after adding a pseudo value of 1.

Gene expression database

Gene expression databases of rectal cancer patient pre-treatment biopsies prior to neoadjuvant CRT were downloaded from the GEO database (http://www.ncbi.nlm.nih.gov/geo/). GSE109057[2] was previously produced by our group using the Affymetrix PrimeView Human Gene Expression Array (Affymetrix, Santa Clara, CA) and a cohort of 90 patients, of whom 81 patients were also analyzed by RNA sequencing in the present study. GSE87211[3] was produced using an Agilent-026652 Whole Human Genome Microarray 4x44K v2 with a cohort of 203 patients, whereas GSE45404[4] was produced using an Affymetrix Human Genome U133 Plus 2.0 Array with a cohort of 42 patients.

© 2023 Akiyoshi T et al. *JAMA Network Open.*

eReferences

1. Comprehensive molecular characterization of human colon and rectal cancer. *Nature.* 2012;487(7407):330-337.
2. Akiyoshi T, Tanaka N, Kiyotani K, et al. Immunogenomic profiles associated with response to neoadjuvant chemoradiotherapy in patients with rectal cancer. *Br J Surg.* 2019;106(10):1381-1392.
3. Hu Y, Gaedcke J, Emons G, et al. Colorectal cancer susceptibility loci as predictive markers of rectal cancer prognosis after surgery. *Genes Chromosomes Cancer.* 2018;57(3):140-149.
4. Agostini M, Zangrando A, Pastrello C, et al. A functional biological network centered on XRCC3: a new possible marker of chemoradiotherapy resistance in rectal cancer patients. *Cancer Biol Ther.* 2015;16(8):1160-1171.

© 2023 Akiyoshi T et al. *JAMA Network Open.*
