# Supplementary material for: Transcriptomic Analyses of Pretreatment Tumor Biopsy Samples, Response to Neoadjuvant Chemoradiotherapy, and Survival in Patients With Advanced Rectal Cancer
Source: JAMA Netw Open. 2023 Jan 20;6(1):e2252140. doi: 10.1001/jamanetworkopen.2022.52140 (PMC9860531; doi:10.1001/jamanetworkopen.2022.52140)
Supplement: Supplement 2. — Data Sharing Statement [file jamanetwopen-e2252140-s002.pdf]

Data Sharing Statement

Akiyoshi. Transcriptomic Analyses of Pretreatment Tumor Biopsy Samples, Response to Neoadjuvant Chemoradiotherapy, and Survival in Patients With Advanced Rectal Cancer. *JAMA Netw Open*. Published January 20, 2023. doi:10.1001/jamanetworkopen.2022.52140

Data
Data available: No
